# [Corrigendum] Role of EphA2-PI3K signaling in vasculogenic mimicry induced by cancer-associated fibroblasts in gastric cancer cells

**DOI:** 10.3892/ol.2026.15496

**Published:** 2026-02-24

**Authors:** Hee Sung Kim, You Jin Won, Ju Hee Shim, Hyun Ji Kim, Byung Sik Kim, Hea Nam Hong

Oncol Lett 18: 3031–3038, 2019; DOI: 10.3892/ol.2019.10677

Subsequently to the publication of this paper, an interested reader drew to the attention of the Editor that, concerning the VM tube formation assay images shown in Figs. 3A and 4A, a number of the images appeared to contain overlapping sections. Specifically, within Fig. 3A, the 48 h/CAF-CM image appeared to be overlapping with the 48 h/CAF-CM/PD image, and the 48 h/CAF-CM image in Fig. 3A was also overlapping with the 48 h/CAF-CM/siCON image in Fig. 4A.

After having re-examined the raw data for these figures, the authors have realized that Figs. 3A and 4A were inadvertently assembled incorrectly. Corrected versions of Figs. 3 and 4, now showing the correct data for the 48 h/CAF-CM/PD panel in Fig. 3A and the 48 h/CAF-CM/siCON panel in Fig. 4A, are shown below and on the next page. The authors regret the errors that were made during the compilation of the original figures, and are grateful to the editor of *Oncology Letters* for allowing them the opportunity to publish a Corrigendum; furthermore, they apologize to the readership for any inconvenience caused.

## Figures and Tables

**Figure 3.**
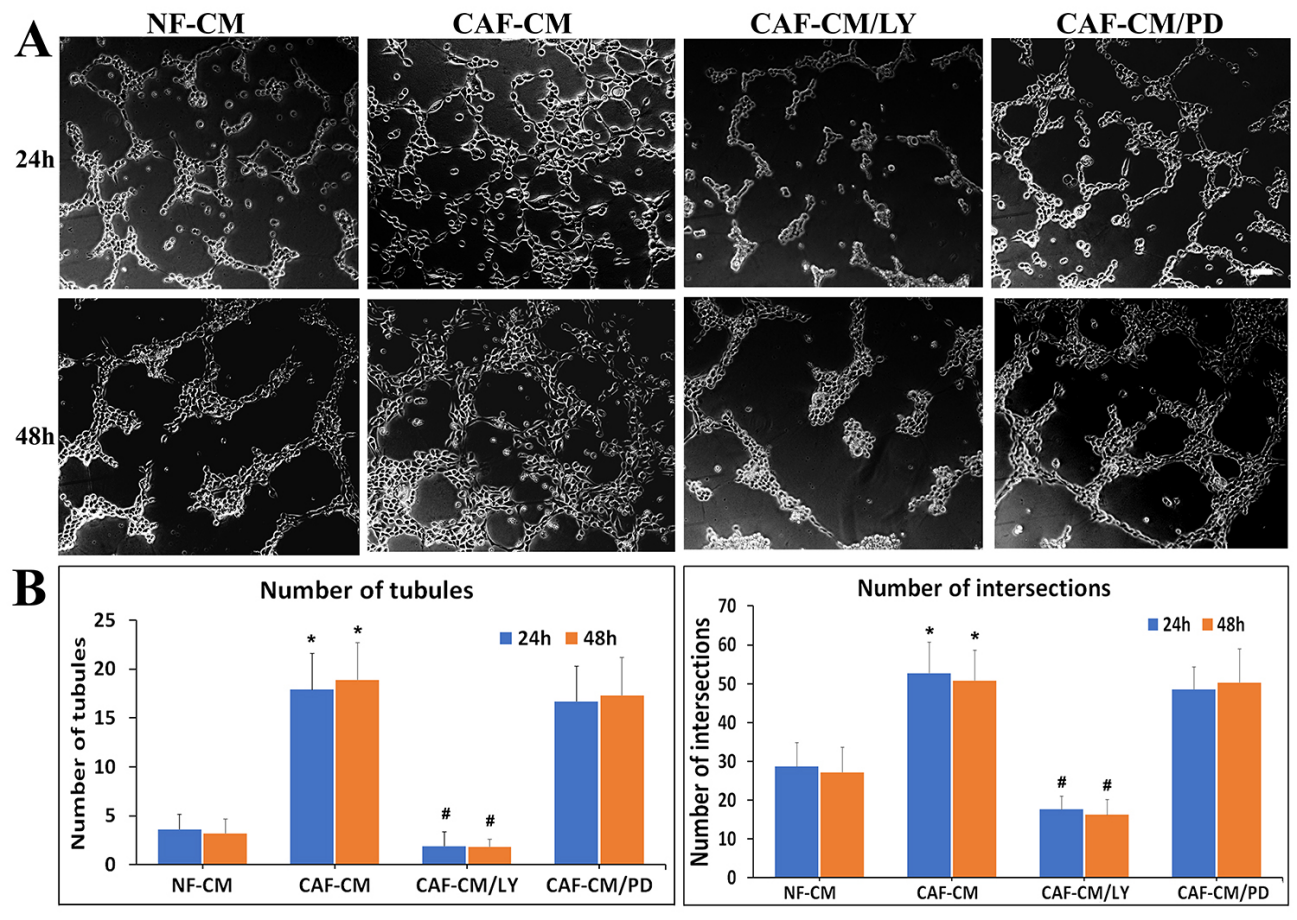
CAF-CM-induced vasculogenic mimicry formation is blocked by a PI3K/AKT-inhibitor in gastric cancer cells. (A) AGS gastric cancer cells were treated with CAF-CM (50%) with or without inhibitors of PI3K/AKT signaling (LY294002, 20 µM) and MAPK-ERK kinase (PD98059, 20 µM) for 24 and 48 h. Scale bars, 100 µm. (B) Graphs present the number of tubules and intersections. Values are expressed as the mean ± standard deviation of three independent experiments. *P<0.001 vs. control; ^#^P<0.001 vs. CAF-CM. CAF, cancer-associated fibroblast; CM, conditioned-medium; PI3K, phosphoinositide 3-kinase; AKT, protein kinase B; MAPK, mitogen activated protein kinase; ERK, extracellular signal-regulated kinases; NF, normal gastric fibroblasts; LY, LY294002; PD, PD98059.

**Figure 4.**
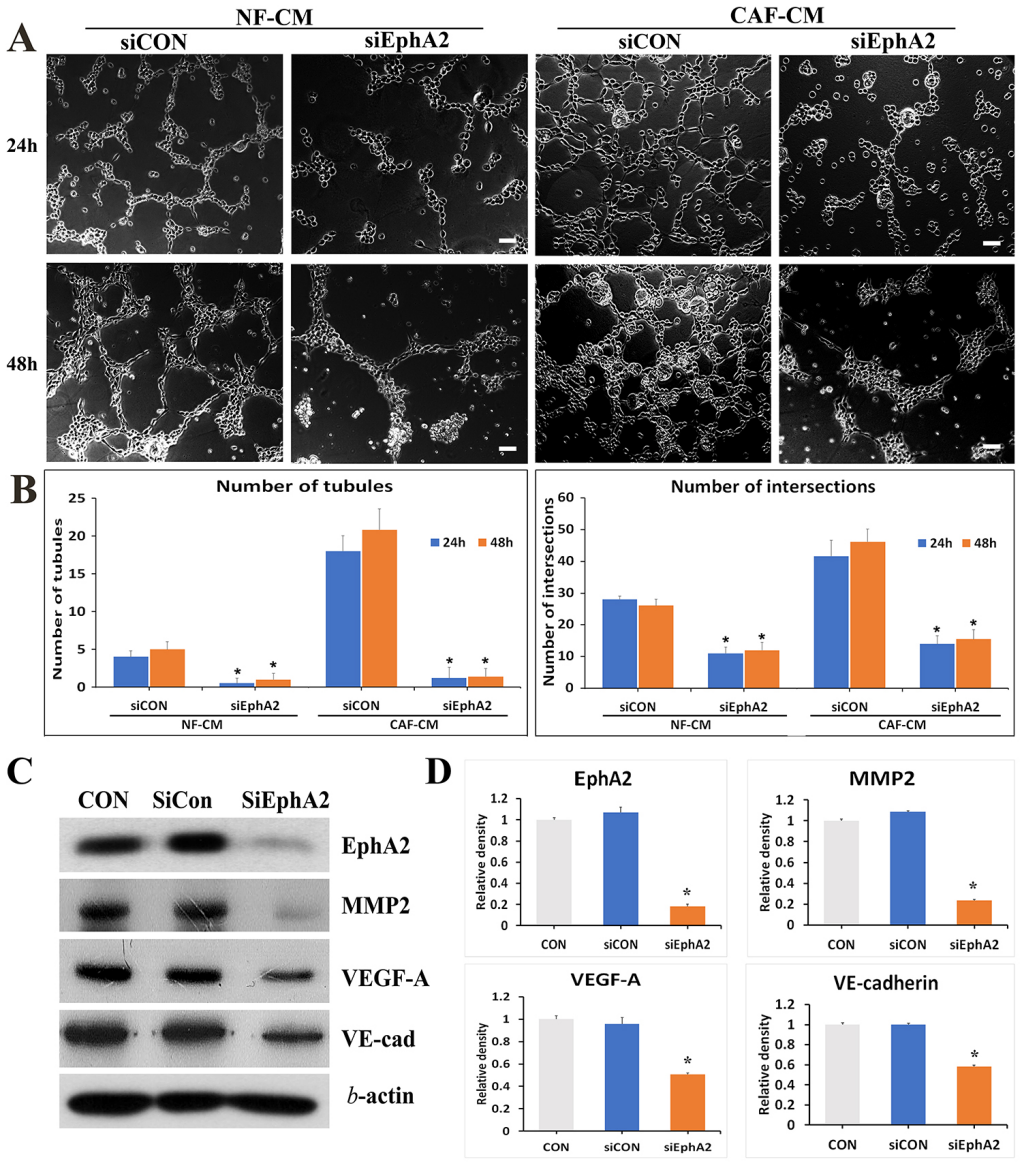
Silencing of the EphA2 gene suppresses the VM forming ability of gastric cancer cells. (A) Representative images of VM formation in AGS cells transfected with siCON or siEphA2 for 24 and 48 h, in either NF-CM or CAF-CM (50%). Scale bars, 100 µm. (B) Graphs present the number of tubules and intersections in each experimental group. (C) Western blot images of EphA2, MMP2, VEGF-A and VE-cadherin expression in siCON and siEphA2 AGS cells. (D) Graphs presenting the protein expression levels of EphA2, MMP2, VEGF-A and VE-cadherin. Mean ± standard deviation from three independent experiments. *P<0.001 vs. siCON. EphA2, erythropoietin-producing human hepatocellular receptor A2; VM, vasculogenic mimicry; CON, non-treated AGS cells; siRNA, small interfering RNA; siCON, non-silencing siRNA; siEphA2, siRNA-EphA2; VE-cad, VE-cadherin; NF, normal gastric fibroblasts; CM, conditioned medium; CAF, cancer-associated fibroblasts; MMP2, matrix metalloproteinase 2; VEGF-A, vascular endothelial growth factor A.

